# Evaluation of Index of Cardiac Electrophysiological Balance in Patients With Myotonic Dystrophy Type 1

**DOI:** 10.7759/cureus.34600

**Published:** 2023-02-03

**Authors:** Metin Okşul, Önder Bilge, Askeri Türken, Ferhat Işık, Abdurrahman Akyüz, Murat Çap, Serdar Söner, Halil Akın, Yusuf Z Şener, Ercan Taştan

**Affiliations:** 1 Department of Cardiology, University of Health Sciences Diyarbakır Gazi Yaşargil Education and Research Hospital, Diyarbakır, TUR; 2 Department of Physical Therapy and Rehabilitation, University of Health Sciences Diyarbakır Gazi Yaşargil Education and Research Hospital, Diyarbakır, TUR; 3 Department of Cardiology, Private Medicalpark Hospital, Ankara, TUR; 4 Department of Cardiology, Beypazari State Hospital, Ankara, TUR

**Keywords:** electrocardiography (ecg), ventricular dysrhythmia, sudden cardiac death, index of cardiac electrophysiological balance, myotonic dystrophy type 1

## Abstract

Background: Myotonic dystrophy type 1(MD1), which is characterized by decreased muscle tone, progressive muscle weakness, and cardiac involvement, is an autosomal dominant and progressive congenital muscle disease. Cardiac involvement more often manifests as conduction abnormalities and arrhythmias (such as supraventricular or ventricular). Approximately one-third of MD1-related deaths occur due to cardiac causes. The index of cardiac-electrophysiological balance (ICEB) is a current parameter calculated as QT interval/QRS duration. The increase in this parameter has been associated with malignant ventricular arrhythmias. In this study, our aim was to compare the ICEB values ​​of MD1 patients and the normal population.

Material and method: A total of 62 patients were included in our study. They were divided into two groups - 32 MD patients and 30 controls. The demographic, clinical, laboratory, and electrocardiographic parameters of the two groups were compared.

Results: The median age of the study population was 24 (20-36 IQR), and 36 (58%) of these patients were female. Body mass index was higher in the control group (p = 0.037). While in the MD1 group creatinine kinase was significantly higher (p <0.001), In the control group creatinine, aspartate aminotransferase, alanine aminotransferase, calcium, and lymphocyte levels were significantly higher (p=0.031, p= 0.003, p=0.001, p=0.002, p=0.031, respectively). ICEB [3.96 (3.65-4.46) vs 3.74 (3.49-3.85) p=0.015] and corrected ICEB (ICEBc) [4.48 (4.08-4.92) vs 4.20 (4.03-4.51) p = 0.048] were significantly higher in the MD1 group.

Conclusion: In our study, ICEB was found to be higher in MD1 patients than in the control group. Increased ICEB and ICEBc values ​​in MD1 patients may precipitate ventricular arrhythmias in the future. Close monitoring of these parameters can be helpful in predicting possible ventricular arrhythmias and in risk stratification.

## Introduction

Myotonic dystrophy (MD) caused by autosomal dominant inheritance is the most common neuromuscular disease in adults. MD type 1 (MD1), also known as Steinert's disease, is the most common of the two types. MD1 has a worse prognosis, and its incidence is thought to be 1 in 8000. This disease is characterized by progressive atrophy and skeletal muscle weakness as well as systemic manifestations such as cardiac involvement and respiratory failure. MD1 is caused by an expanded cytosine- thymine-guanine (CTG) repeat on chromosome 19 in the 3′ untranslated region of a serine-threonine protein kinase gene called DMPK (dystrophia myotonica protein kinase) [1,2].

 The main cause of death in MD1 patients is respiratory failure, and the second most common cause is due to cardiac involvement. Sudden cardiac death is not rare in this patient group and is often attributed to cardiac arrhythmias secondary to myocardial fibrosis. Cardiac manifestations can be observed in a spectrum ranging from asymptomatic ECG changes to ventricular fibrillation [3].

The index of cardiac electrophysiological balance (ICEB) is a non-invasive indicator that has been used more frequently recently and is a predictor of cardiac arrhythmias. ICEB is obtained by dividing the QT interval by the QRS duration on surface electrocardiography (ECG). Any deviation of this value from normal was found to be predictive of arrhythmias. ICEB is useful in clinical practice as it can be easily measured from the ECG [4]. The aim of this study was to compare the ICEB values ​​of MD1 patients to the control group.

## Materials and methods

In this single-center retrospective study, 30 control patients and 32 MD1 outpatients who were followed up at the physical therapy and rehabilitation department of the Gazi Yaşargil Training and Research Hospital, and who were diagnosed with genetic testing, between March 2019 to September 2019, were enrolled. Patients with a previous diagnosis of arrhythmia and/or using antiarrhythmic medications were excluded from the study.

Electrocardiographic analysis

All patients' 12-lead ECGs (Schiller, Germany Bavaria) were taken in the supine position with a standard speed of 25 mm/s and a calibration of 10 mm/mV. Basal ECGs evaluated in sinus rhythm. DII and V5 leads were used for measurements. Heart rate, QRS duration, QT intervals, corrected QT intervals (QTc), QT/QRS ratio (ICEB), and QTc/QRS ratio (ICEBc) were evaluated by two cardiologists.

Statistical analysis

The IBM SPSS software package was used for all statistical analyzes (IBM SPSS Statistics for Windows, Version 24.0, IBM Corp., Armonk, NY). The normal distribution of the data was evaluated with the Kolmogorov-Smirnov test. The numeric variables showing normal distribution were presented as the mean ± standard deviation, and the numerical variables showing non-normal distribution were presented as the median. The categorical variables were expressed as percentages and numbers. The T-test was used for independent samples in the analysis of numerical variables with normal distribution, and the Mann-Whitney U test was used in the analysis of numerical variables with non-normal distribution to compare two groups. The Chi-square test and Fisher's exact Chi-square test were used to compare categorical data. Values of p < 0.05 were considered to be statistically significant.

## Results

A total of 62 patients were included in our study. They were divided into two groups 32 MD1 patients and 30 controls. 56% of MD1 patients and 60% of the control group were female. The median age of MD1 patients was 28 (20-38) and the median age of the control group was 23 (20-36) (p=0.540). There was no significant difference between the mean age and gender of the two groups. Body mass index was significantly lower in patients with MD1 (22.3 vs 23.7, p=0.037). In the control group creatinine, aspartate aminotransferase, alanine aminotransferase, calcium, and lymphocyte levels were significantly higher (p=0.031, p= 0.003, p=0.001, p=0.002, p=0.031, respectively). Also, creatinine kinase (CK) values ​​of MD1 patients were significantly higher than the control group (223 vs. 110, p<0.001). There was no significant difference between the sodium and potassium values ​​of the patients in the two groups. Although the platelet count was higher in the MD1 group, it was not statistically significant (p=0.056). The baseline characteristics and laboratory parameters of the patients are summarized in Table 1.

**Table 1:** The demographic, clinical characteristics, and laboratory of the study population

Parameters	All patients (n=62)	MD1 group (n= 32)	Control group (n=30)	P-value
Age, year	24 (20-36)	28 (20-38)	23 (20-36)	0.540
Gender female, n (%)	36 (58)	18 (56)	18 (60)	0.802
Body mass index, kg/m^2^	23.6 (20.2-25.4)	22.3 (19.4-24.0)	23.7 (20.0-25.4)	0.037
Alanin Aminotransferase, IU/L	25 (16-30)	17 (13-23)	27 (25-34)	0.001
Aspartat Aminotransferase, IU/L	24 (17-29)	18 (26-15)	27 (22-29)	0.003
Creatinine, mg/dL	0.71 (0.61-0.82)	0.66 (0.57-0.79)	0.73 (0.67-0. 85)	0.031
Creatine kinase, mg/dL	126 (95-231)	223 (106-274)	110 (79-127)	< 0.001
Calsiyum, mg/dL	9.5 (9.2-10.0)	9.3 (9.0-9.7)	9.8 (9.4-10.1)	0.002
Sodium, mmol/L	140 (138-141)	139 (138-141)	140 (138-141)	0.752
Potassium, mmol/L	4.3 (4.0-4.5)	4.1 (4.0-4.4)	4.3 (4.1-4.6)	0.109
White blood cell, 10^3^/µL	7.94 (6.64-9.45)	7.87 (6.51-9.45)	8.37 (7.14-9.45)	0.741
Neutrophil, 10^3^/µL	4.73 (3.80-6.41)	4.94 (3.70-6.70)	4.66 (3.94-6.41)	0.866
Lymphocyte, 10^3^/µL	2.38 (1.82-2.60)	2.12 (1.73-2.54)	2.50 (2.03-2.76)	0.031
Hemoglobin, gr/dL	14.3 (13.2-14.9)	14.4 (13.0-14.9)	13.9 (13.2-15.0)	0.821
Platelet, 10^9^/L	278 (253-332)	302 (262-355)	266 (245-313)	0.056

There was no statistically significant difference between the two groups' heart rate, PR interval, QT interval, corrected QT interval, and QRS durations (p values ​​0.256, 0.928, 0.080, 0.506, 0.166, respectively). ICEB and ICEBc values ​​of MD patients were significantly higher than the control group (p-values ​​0.01 and 0.048, respectively). The electrocardiographic finding of the study population summarized in Table 2.

**Table 2:** The electrocardiographic finding of the study population ICEB: Index of cardiac electrophysiological balance, ICEBc: Corrected index of cardiac electrophysiological balance

Parameters	All patients (n=62)	MD1 group (n= 32)	Control group (n=30)	P-value
PR interval, msn	138 (127-153)	138 (122-157)	137 (127-157)	0.928
Heart rate, bpm	78 (73-86)	77 (72-84)	79 (73-90)	0.256
QT interval, msn	345 (330-359)	349 (340-363)	342 (327-354)	0.080
Corrected QT interval, ms	392 (382-401)	393 (386-400)	390 (382-401)	0.506
QRS duration, msn	90 (84-97)	90 (78-95)	93 (84-98)	0.166
ICEB (QT/QRS)	3.79 (3.57-4.04)	3.96 (3.65-4.46)	3.74 (3.49-3.85)	0.015
ICEBc (QTc/QRS)	4.33 (4.06-4.70)	4.48 (4.08-4.92)	4.20 (4.03-4.51)	0.048

## Discussion

To the best of our knowledge, this is the first study to investigate ICEB and ICEBc in MD1 patients. The results of this study showed that ICEB and ICEBc values ​​were higher in MD1 patients than in the control group.

MD1 disease is an autosomal dominant, multisystemic genetic disease that affects the skeletal, cardiac, and smooth muscles as well as the brain, lens, and endocrine systems. MDI is caused by unstable microsatellite (CTG)nexpansion (n>50) in the 30 untranslated regions of the DMPKgene. Sudden death is common as a result of respiratory failure secondary to the involvement of respiratory muscles. In these patients, Cardiac-related deaths are the second most common cause of sudden death. The most common ECG abnormality reported in MD1 patients is first-degree atrioventricular block. According to a meta-analysis, its incidence is reported to be between 25 and 45%. Other ECG abnormalities, which occur in approximately 15-20% of cases, are bundle branch blocks and/or QRS prolongation (>120 ms), atrial fibrillation, and atrial flutter. It has been reported that atrial fibrillation and atrial flutter were observed in the range of 8-22% in 24-hour Holter monitoring. It may also present with other rare cardiac manifestations such as sudden death, heart failure, Brugada syndrome, ischemic heart disease, mitral valve prolapses and rarely dilated cardiomyopathy. In these patients, atrial fibrillation and other arrhythmias increase the risk of cerebral ischemia, and mortality and morbidity vary in relation to cardiac early diagnosis and treatment. Patients who have cardiac involvement secondary to MD1 may experience palpitations, dizziness, fatigue, or sometimes syncope, but are often asymptomatic [1,2,5,6]. It has been reported that the annual incidence of sudden death is between 0.53 to 1.6% in these patients. It is thought that complete AV block and subsequent asystole and ventricular arrhythmias due to cardiac conduction defect are the main abnormalities causing sudden cardiac death. Previous studies have indicated that severe ECG abnormalities determined in these patients are an independent predictor of sudden cardiac death [7-9].

ICEB is a parameter based on measuring the balance between depolarization (QRS) and repolarization (QT) of the ventricle. This parameter was used for the first time in the literature to predict drug-induced ventricular arrhythmias. In a study with rabbit hearts conducted by Lu et al., deviation of this balance from normal has significant value in predicting drug-induced arrhythmias. In this study, it has been determined that the increase of ICEB was associated with ventricular conduction slowdown, QT shortening, and an increase in the frequency of non-torsades-d-pointes (TdP) ventricular tachycardia/ ventricular fibrillation [10]. In a study conducted by Robyns et al., which is based on an electrophysiological basis of 40 patients, have been found that increased ICEB was a good predictor of ventricular arrhythmias [4].

The results of large studies and data from meta-analyses indicate that one of the most common causes of sudden cardiac death in MD1 patients is ventricular arrhythmias. It is thought that the main reason underlying ventricular arrhythmias is conduction defects due to subendocardial fibrosis, and substrate formation in the ventricular myocardium that predisposes to reentry. In these patients, although non-sustained VT is a predictor for sustained VT, in some patients, sustained ventricular tachycardia may develop as the first attack. However, it is not yet known exactly which patients are at higher risk for sudden cardiac death [2,11,12].

In 1988, Nguyen et al. published the first detailed autopsy reports of 12 patients with myotonic dystrophy. In this series of these patients, it was determined that the most common pathological findings in myocardial samples were fibrosis, fat infiltration, and atrophy in the conduction system. In most of these cases, despite the absence of any cardiac symptoms before death, some had ECG abnormalities [13]. In the literature, many studies have been conducted on these patients for risk classification. In MD1 patients, despite all this, electrocardiographic predictors of sudden death and which patients are at risk for sudden cardiac death are still controversial issues [8].

Sudden death due to ventricular arrhythmias is a global problem not only for MD1 patients but also for many diseases with cardiac involvement. And which patients are more at risk for ventricular arrhythmias is one of the most important areas of cardiology. Identification of high-risk patients is a guide for many preventive treatments (such as implantable cardioverter defibrillators and anti-arrhythmic drugs) for the prevention of sudden cardiac death. Many electrocardiographic parameters have been investigated to determine the risk of ventricular arrhythmia. These are corrected QT (QTc) interval, QT dispersion (QTD), the interval from the peak to the end of the T-wave (Tpeak - Tend), (Tpeak - Tend)/QT, T-wave alternans (TWA), and microvolt TWA. Although QT duration is a good predictor for ventricular arrhythmias, its most important limitation is that it is affected by heart rate. Novel conduction-repolarization markers incorporating λ include Lu et al.’ index of cardiac electrophysiological balance (ICEB: QT/QRS duration) [11,14-16]. Recent studies have shown that increased ICEB, with or without cardiac involvement, maybe a good predictor for the development of ventricular arrhythmias [17,18]. The most important advantage of ICEB is non-invasive, inexpensive, and practical.

According to the results of our study, the increase in ICEB measurement in MD1 patients compared to control patients suggests that this parameter may be a guide for risk assessment in these patients. However, our study has some limitations. First, the study was retrospective, single-center, and was conducted with relatively few patients. Taking into account that MD1 is a very rare disease, it can be considered that the number of patients is sufficient. Secondly, although results based only on electrocardiographic measurements were obtained, there is no information about whether these patients experienced ventricular arrhythmia during the follow-up. Arrhythmic events could be followed better if patients were evaluated with a scanning method such as Holter ECG or implantable loop recorder (ILR).

## Conclusions

In our study, as a result, we found that ICEB and ICEBc values ​​were significantly increased in MD1 patients compared to the control group. And ICEB measurements may be useful in risk classification in these patients There is a need for prospective studies involving larger numbers of patients and also evaluating whether arrhythmic events have occurred.

## References

[1] Groh WJ, Groh MR, Saha C (2008). Electrocardiographic abnormalities and sudden death in myotonic dystrophy type 1. N Engl J Med.

[2] Wahbi K, Babuty D, Probst V (2017). Incidence and predictors of sudden death, major conduction defects and sustained ventricular tachyarrhythmias in 1388 patients with myotonic dystrophy type 1. Eur Heart J.

[3] Mahadevan MS, Yadava RS, Mandal M (2021). Cardiac pathology in myotonic dystrophy type 1. Int J Mol Sci.

[4] Robyns T, Lu HR, Gallacher DJ (2016). Evaluation of index of cardio-electrophysiological balance (ICEB) as a new biomarker for the identification of patients at increased arrhythmic risk. Ann Noninvasive Electrocardiol.

[5] Wenninger S, Montagnese F, Schoser B (2018). Core clinical phenotypes in myotonic dystrophies. Front Neurol.

[6] Chou CC, Chang PC, Wei YC, Lee KY (2017). Optical mapping approaches on muscleblind-like compound knockout mice for understanding mechanistic insights into ventricular arrhythmias in myotonic dystrophy. J Am Heart Assoc.

[7] Petri H, Vissing J, Witting N, Bundgaard H, Køber L (2012). Cardiac manifestations of myotonic dystrophy type 1. Int J Cardiol.

[8] Wahbi K, Meune C, Porcher R (2012). Electrophysiological study with prophylactic pacing and survival in adults with myotonic dystrophy and conduction system disease. JAMA.

[9] Petri H, Witting N, Ersbøll MK (2014). High prevalence of cardiac involvement in patients with myotonic dystrophy type 1: a cross-sectional study. Int J Cardiol.

[10] Wahbi K, Furling D (2020). Cardiovascular manifestations of myotonic dystrophy. Trends Cardiovasc Med.

[11] Lu HR, Yan GX, Gallacher DJ (2013). A new biomarker--index of cardiac electrophysiological balance (iCEB)--plays an important role in drug-induced cardiac arrhythmias: beyond QT-prolongation and Torsades de Pointes (TdPs). J Pharmacol Toxicol Methods.

[12] Merino JL, Carmona JR, Fernández-Lozano I, Peinado R, Basterra N, Sobrino JA (1998). Mechanisms of sustained ventricular tachycardia in myotonic dystrophy: implications for catheter ablation. Circulation.

[13] Nguyen HH, Wolfe JT 3rd, Holmes DR Jr (1988). Pathology of the cardiac conduction system in myotonic dystrophy: a study of 12 cases. J Am Coll Cardiol.

[14] Rakocevic Stojanovic V, Peric S, Paunic T (2013). Cardiologic predictors of sudden death in patients with myotonic dystrophy type 1. J Clin Neurosci.

[15] Priori SG, Blomström-Lundqvist C, Mazzanti A (2015). 2015 ESC Guidelines for the management of patients with ventricular arrhythmias and the prevention of sudden cardiac death: The Task Force for the Management of Patients with Ventricular Arrhythmias and the Prevention of Sudden Cardiac Death of the European Society of Cardiology (ESC). Endorsed by: Association for European Paediatric and Congenital Cardiology (AEPC). Eur Heart J.

[16] Tse G, Yan BP (2017). Traditional and novel electrocardiographic conduction and repolarization markers of sudden cardiac death. Europace.

[17] Afsin A, Asoglu R, Kobat MA, Asoglu E, Suner A (2021). Evaluation of index of cardio-electrophysiological balance in patients with atrial fibrillation on antiarrhythmic-drug therapy. Cardiol Res.

[18] Sivri S, Çelik M (2019). Evaluation of index of cardiac-electrophysiological balance before and after hemodialysis in patients with end-stage renal disease. J Electrocardiol.

